# Commentary: Handwriting but not typewriting leads to widespread brain connectivity: a high-density EEG study with implications for the classroom

**DOI:** 10.3389/fpsyg.2024.1517235

**Published:** 2025-01-08

**Authors:** Svetlana Pinet, Marieke Longcamp

**Affiliations:** ^1^Basque Center on Cognition, Brain and Language, Donostia-San Sebastián, Spain; ^2^Ikerbasque, Basque Foundation for Science, Bilbao, Spain; ^3^Aix Marseille Univ, CNRS, CRPN, Marseille, France

**Keywords:** learning, typing, handwriting, theta/alpha oscillations, EEG

## Introduction

Van Der Weel and Van Der Meer (2024, hereafter VWVM2024) claims that, unlike typing, handwriting generates brain connectivity patterns that promote learning. This result leads the authors to stress “the urge that children, from an early age, must be exposed to handwriting activities in school.” This study had a broad impact in the scientific community and in the media: 84,680 views and 11,150 downloads, relayed by 179 news outlets, and tweeted by 894^1^ (for reference, other research articles published in the same month averaged 1,000–4,000 views). Despite the relevance of the topic addressed, we point to limitations in the protocol, analysis, and interpretations of the results that cast some doubts about the validity of the conclusions.

## Weak evidence for an effect on learning

VWVM2024 states: “As increased connectivity in the brain was observed only when writing by hand and not when simply pressing keys on the keyboard, our findings can be taken as evidence that handwriting promotes learning” (p. 7). Their rationale is that since connectivity during handwriting is “far more elaborate” (p. 1), handwriting facilitates learning and should be practiced from a young age at school. This logical shortcut deserves scrutiny, for two reasons.

First, the protocol did not include any learning. Participants repeatedly wrote well-known words without any requirement for memory encoding, preventing any conclusions in terms of learning. Moreover, VMVW2024 is a lab-based study with an adult population. Translation from well-controlled protocols to educational settings in a child population is not straightforward. The very possibility of using research from fundamental cognitive neuroscience to inform educational practices is debated (Bowers, 2016; Gabrieli, 2016; Horvath and Donoghue, 2016). In sum, drawing conclusions on learning processes in children in a classroom from a lab study carried out on a group of university students that did not include any type of learning seems slippery at best.

Second, the interpretation of increased theta/alpha connectivity as an unequivocal indicator of a brain state favorable to learning and remembering is problematic. While theta and alpha oscillations have been functionally related to a variety of cognitive processes (Bastiaansen et al., 2008; Brier et al., 2010; Cavanagh and Frank, 2014; Michelmann et al., 2022), it has not been clearly established that increased theta/alpha connectivity creates appropriate conditions for learning. Among the studies cited by the authors to support their claim, Solomon et al. (2017) indeed reported increased theta connectivity, but in situations of explicit encoding and retrieval (Andres et al., 1999 for a similar finding in alpha/beta frequency bands). Raghavachari et al. (2001) showed increased theta oscillations in a working memory task. It remains a stretch to use this finding as proof that theta connectivity promotes learning; handwriting might simply require more sustained working memory maintenance because it is generally slower than typing.

## Claims unsupported by the results

VWVM2024 makes strong claims such as “Handwriting but not typewriting leads to widespread brain connectivity” (p. 1). However, only the difference between handwriting and typing is reported in the results, not connectivity patterns for each condition separately. While Figure 2 of VWVM2024 displays coherence plots for each condition, statistical analysis was performed exclusively on the difference between handwriting and typing, which precludes any conclusion from being drawn on either condition on its own and puts the validity of the title of the article into question.

## Artificial typing conditions

In VWVM2024, participants were instructed to type using only their right index finger, making the typing condition quite different from typical typing. Skilled typing is characterized by the coordination between hands and the use of several fingers (Feit et al., 2016; Logan et al., 2016). Changing habitual typing behavior to conform to the instructions might impact participants' typing performance and associated brain activity through disruption of automatized control (Logan and Crump, 2009). In addition, imposing a unimanual behavior might have led to artificially decreasing connectivity patterns in typing. Bimanual activities such as typing are associated with regulation of activation/inhibition patterns over both hemispheres (Pinet et al., 2015, 2019) and with increases in inter- and intra-hemispheric connectivity (Andres et al., 1999; Swinnen, 2002). Another important difference between VWVM2024's typing and handwriting conditions is the absence of visual feedback on the screen during typing. Removal of visual feedback decreases typing speed and impairs error monitoring processes (Pinet and Nozari, 2021). In contrast, handwriting was performed under typical conditions (although see Guilbert et al., 2019 for disrupted control of handwriting on a tablet surface).

## Lack of behavioral measures

“The writings produced by the participants […] were stored for offline analyses” (p. 2). Yet, behavioral measures are not reported. Establishing a solid behavioral pattern is usually a requirement before interpreting neurophysiological measures. In VWVM2024, differences in connectivity could have occurred because of differences in the timing and accuracy of handwriting and typing. Moreover, participants' typing skills were neither assessed nor controlled. There is a strong variability in typing skills (Pinet et al., 2022), and non-fluent typing is costly in terms of cognitive resources (Bouriga and Olive, 2021; Graham et al., 2000). Devoting resources to motor processes, at least for less skilled participants, is another factor that could influence brain networks connectivity.

## Conclusion

In conclusion, handwriting and typing are both complex activities, likely to display as many similarities as differences. VWVM2024 attempted to characterize the brain activity patterns associated with each modality, an undoubtedly challenging endeavor. However, implications of their findings for learning would be more convincing if limitations in the experimental protocol, reporting of the results, and interpretations were addressed.

We wish to stress that putting into question VWVM2024′s conclusions should not be taken as putting into question the importance of handwriting. Previous evidence does support a beneficial role of handwriting training on single letter recognition (James and Engelhardt, 2012; Longcamp et al., 2005), word recall (Mangen et al., 2015) and word reading and writing (Mangen and Velay, 2010), although long-term consequences remain to be evaluated. The substantial media coverage received by VWVM2024 shows the public interest for this topic as part of the timely issue of how technological development shapes learning and education. We fully agree with VWVM2024 on the urge to examine the implications of writing practice for learning and memory, at a time when handwriting is receiving less attention. Studies including basic research, interventional applied research with converging conclusions, and scaling to the classroom are needed to settle this question and eventually inflect educational policies.

## Footnotes

^1^According to Frontiers Publication impact at the time of submission (October, 25th 2024).
